# Presence of Contagious Yawning in Children with Autism Spectrum Disorder

**DOI:** 10.1155/2013/971686

**Published:** 2013-07-22

**Authors:** Saori Usui, Atsushi Senju, Yukiko Kikuchi, Hironori Akechi, Yoshikuni Tojo, Hiroo Osanai, Toshikazu Hasegawa

**Affiliations:** ^1^Department of Cognitive and Behavioral Science, University of Tokyo, Tokyo, Japan; ^2^Centre for Brain and Cognitive Development, Birkbeck, University of London, Malet Street, London WC1E 7HX, UK; ^3^Department of Education for Children with Disabilities, Ibaraki University, Ibaraki, Japan; ^4^Musashino Higashi Center for Education and Research, Musashino Higashi Gakuen, Tokyo, Japan

## Abstract

Most previous studies suggest diminished susceptibility to contagious yawning in children with autism spectrum disorder (ASD). However, it could be driven by their atypical attention to the face. To test this hypothesis, children with ASD and typically developing (TD) children were shown yawning and control movies. To ensure participants' attention to the face, an eye tracker controlled the onset of the yawning and control stimuli. Results demonstrated that both TD children and children with ASD yawned more frequently when they watched the yawning stimuli than the control stimuli. It is suggested therefore that the absence of contagious yawning in children with ASD, as reported in previous studies, might relate to their weaker tendency to spontaneously attend to others' faces.

## 1. Introduction

Yawning is widespread among vertebrate species, including a wide range of mammals [1]. In humans, yawning is detectable even in the foetus [2]. The function of yawning is still unclear, but a recent theory highlighted that it may have a communicative function [3]. This hypothesis suggests that yawning is a nonverbal form of communication that synchronizes the behavior of a group [4–7]. It has been suggested that yawning transmits physiological and psychological states, such as drowsiness [8, 9], boredom [10], hunger, and mild psychological stress [7], to other members of the group. Among the evidence that supports this theory, studies of contagious yawning have attracted the most attention in recent years.

 Contagious yawning, in which observation of another's yawn induces yawning behaviour in the observer, is a well-documented phenomenon. In humans, contagious yawning can be elicited by viewing or hearing others' yawning or imagining yawning (e.g., [5, 11–14]). During the course of development, contagious yawning can be reliably observed by around 4 to 6 years of age [15, 16] but might not be present in younger infants and toddlers [17]. The presence of contagious yawning has also been reported in several nonhuman animals (e.g., [18–20]). Several neuroimaging studies have been conducted to investigate the cortical and subcortical structures relevant to the contagious yawning [21–24], but the results are inconsistent. This is possibly due to differences in the yawning stimuli and/or control stimuli used for recording [25].

To date, three independent studies have consistently demonstrated the absence of contagious yawning in individuals with autism spectrum disorder (ASD), a developmental disorder with profound impairments in social interaction and communication [26]. First, Senju et al. [27] presented video clips of yawning faces, as well as faces demonstrating mouth-opening actions, the latter serving as control stimuli. TD children yawned more during or after observing yawn video clips than during or after control video clips, while the type of video clips observed did not affect the amount of yawning in children with ASD. Second, Giganti and Esposito Ziello [12] reported the absence of contagious yawning in children with ASD when seeing or listening to others' yawning, even though these children demonstrated the same frequency of spontaneous yawns as control children. Third, Helt et al. [16], using live yawning stimuli, reported less susceptibility to contagious yawning in children with ASD compared with control children. These results are interpreted as a manifestation of the impairment in “empathy” in this population. Based on this interpretation, the absence of contagious yawning in ASD results from the difficulty in empathizing with a yawning person.

However, these studies cannot rule out the possibility that individuals with ASD failed to show contagious yawning in previous experiments because of the absence of spontaneous attention to others' faces [28–30]. The possibility of this was discussed even in some of these initial reports of the absence of contagious yawning in ASD [16, 27]. To test this hypothesis, Senju et al. [31] studied whether children with ASD “catch” yawns when their attention is navigated to yawning faces. The study used exactly the same experimental design as Senju et al. [27], except that a small cartoon animation was presented for 1 s in the location where the eyes of the face stimuli would appear, just before the presentation of each face, and children were instructed to fixate on the animation. Both TD children and children with ASD were found to yawn equally frequently in response to the yawning stimuli. However, as both groups also yawned as frequently in response to the control (i.e., nonyawning) stimuli as to the yawning stimuli, it remained open to an alternative interpretation, that controlled fixation on the face might modulate the frequency of spontaneous yawning irrelevant to the perception of others' yawns, not the contagious yawning.

The aim of the current study is to test the presence of contagious yawning in ASD, when children's attention is navigated to yawning stimuli. To achieve that, we designed a gaze-contingent stimulus display, in which the participants' gaze was monitored with an eye tracker, and the yawning and control movies started only when participants were fixating on the eyes (Experiment 1) or the mouths (Experiment 2) of the stimuli. We also asked participants to count the number of people wearing eyeglasses (Experiment 1) or having a beard (Experiment 2), to further ensure that they were attending to the face. We adopted a block design with an interval between blocks, instead of the event-related design with a 1-minute interstimulus interval used in Senju et al. [27] and Senju et al. [31]. This was to prevent any possible long-latency effects of the yawning stimuli carried over to control stimuli, which might have affected previous results. Other studies adopting block designs have demonstrated clearer effects of yawning stimuli (e.g., [12]). 

Three alternative predictions can be derived from different hypotheses. Firstly, if individuals with ASD have an inherent impairment in empathizing which impedes contagious yawning, we should not observe contagious yawning (i.e., an increase in participants' yawning in response to the observation of yawning stimuli). Secondly, if atypical attention to the face is relevant to the absence of contagious yawning in individuals with ASD, they should show contagious yawning when presentation of yawning stimuli is contingent on their attention to the face. Thirdly, if the fixations on the eyes have a critical role in the processing of yawning face [14] or attentional engagement to the face [29] in individuals with ASD, we should only see the contagious yawning when their attention is navigated to the eyes (Experiment 1), but not to the mouth (Experiment 2). Further, as previous studies have reported that children with ASD show equally frequent spontaneous yawning as TD children [12, 27, 31], we predict that there should be no difference between groups in the number of yawns in the control condition.

## 2. Experiment 1

### 2.1. Method

#### 2.1.1. Participants

The data of 46 TD participants (25 males and 21 females) and 26 participants with ASD (20 males and 6 females) were included in the final analyses (Table 1).

Additional four participants were tested but not included in the analyses because their eye-tracking data was not recorded. This was either due to eye tracker problems or not providing valid gaze data in 50% or more observations during the period of stimulus presentation in a yawn block (a male participant with ASD and 3 TD male participants [32–34]). Moreover, an additional male participant with ASD was tested but not included in the analyses because he was unable to answer the question about what the models were doing after the yawning block [11].

Most of the children were recruited from Musashino Higashi Gakuen which has a program to educate TD children and children with ASD. All the children with ASD had been diagnosed with autism (*N* = 14) or pervasive developmental disorder (*N* = 12) by at least one child psychiatrist, clinical psychologist, or pediatrician before they participated in this experiment. The participants' parents all completed the Japanese version of the Autism Screening Questionnaire (ASQ-J [35, 36]). All ASQ-J scores of the TD children were below the cut-off point (13), and those of the children with ASD were at or above the cut-off point.

An abbreviated version (2 subsets; Picture Completion and Information) of the Japanese WISC-III (WISC-III [37, 38]) was administered to the children under 17 years old, and that of the Japanese WAIS-R [39, 40] was administered to the children aged 17 and over, to measure their IQ. There was no significant difference between TD children and children with ASD in chronological age (*t* = 1.55, *P* = .13, *r* = .18). The mean IQ of the TD children, however, was significantly higher than that of the children with ASD (*t*(31.2) = −2.63 *P* < .05, *r* = .43). There was no significant difference between TD children and children with ASD in sex ratio (*X* ^2^(1) = 3.61, *P* < .10, *φ* = −.22). As IQ or gender did not affect the frequency of contagious yawning in the previous study [27], we matched groups by chronological age. However, the potential effects of age, IQ, and gender were also analyzed.

#### 2.1.2. Stimuli

**Figure 1:**
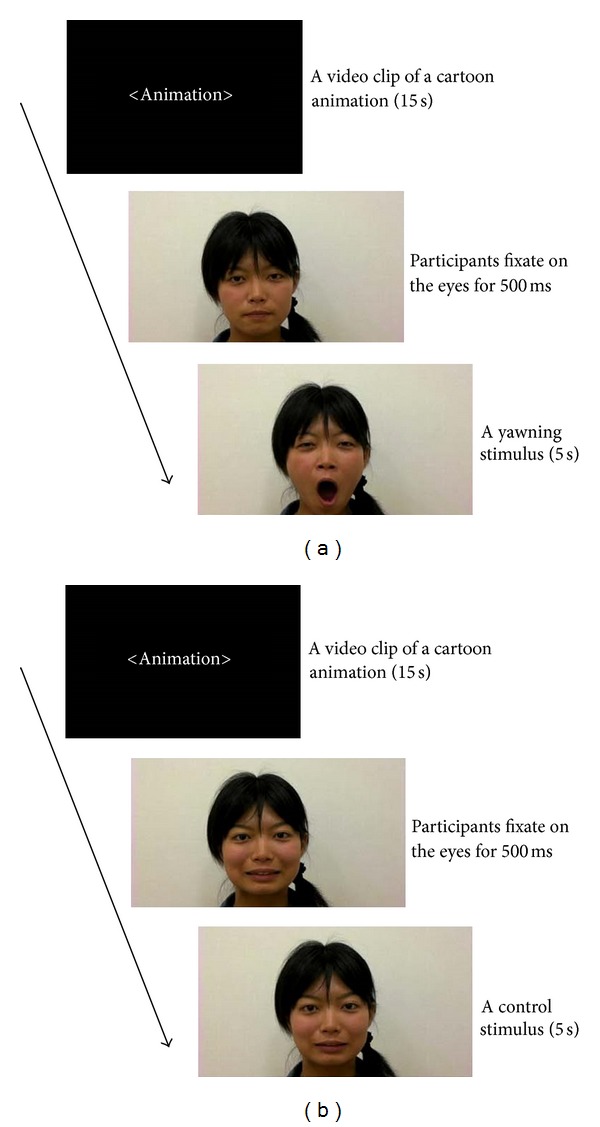
An example of the stimulus sequence of (a) yawn block and (b) control block. This sequence was repeated 24 times, and a video clip of a cartoon animation was presented at the end in each block.

 The stimuli consisted of 12 video clips of yawning faces (5 s each, Figure 1(a)) taken from different adult models and 12 control video clips (widthwise mouth opening, Figure 1(b), 5 s each) of the same models. The gaze of the models was straight ahead except in the yawn condition, in which the eyes of the models were closed briefly during the yawning reflex. All the models were unfamiliar to the participants. The video clips (15 s each) of a silent cartoon animation were presented to focus the participants' attention on the display at the beginning, the end, and during the intervals.

 The experiment consisted of 2 blocks. The video clips of a cartoon animation and yawning faces were alternately presented in one block, and the video clips of a cartoon animation and mouth-opening faces were alternately presented in the other block. The order of the blocks was counterbalanced between the participants, and the interval between the blocks was more than 30 minutes. The stimuli were presented 24 times (12 video clips, twice each) for each block in a pseudorandom order.

**Figure 2:**
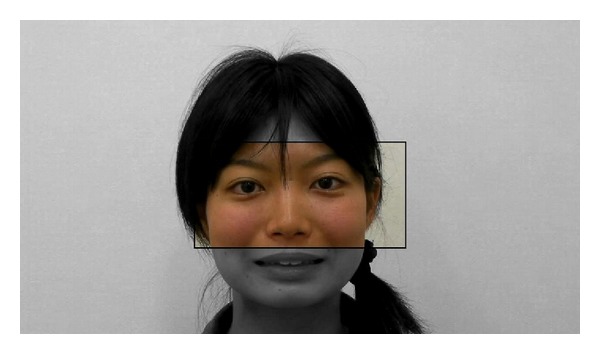
The eye region of the stimuli: the stimuli started only when participants fixate within the black frame for at least 500 ms in Experiment 1.

 Stimulus sequences were presented on an LCD monitor integrated with an eye tracker (Tobii 2150), which was placed about 60 cm from the participant. The eye tracker controlled the onset of stimuli (i.e., video clips of yawning and mouth-opening faces), which started only when participants fixated on the area around the eyes of the model for at least 500 ms (Figure 1). The face that appeared was oriented 25° vertically and 20° horizontally, and the region of the eyes was oriented 10° vertically and 20° horizontally (Figure 2), and the viewing distance was 60 cm. We also recorded the eye-tracking data during the stimulus presentation (see Electronic Supplementary Material for details available online at http://dx.doi.org/10.1155/2013/971686).

#### 2.1.3. Procedure

 All participants viewed the movies in a soundproofed room between about 9 : 30 and 18 : 30. We conducted a 5-point calibration for eye tracking before each block. The fixation radius was 30 pixels, and the minimum fixation duration was 100 ms. They were asked to relax as if they were at home and to fixate on the eyes whenever faces appeared on the screen. They were also asked to watch the movies while leaning back in the chair and to not move the chair, in order to minimize head movements, which was essential for successful eye-tracking. The participants were asked to count the number of times people with glasses appeared, in order to further ensure their attention was drawn to the eyes. All participants viewed the movies by themselves, and an experimenter monitored the participant from another room with a hidden video camera in the testing room. If a participant moved to the extent that it interfered with eye-tracking, an experimenter entered the room, adjusted the head position of the participant and repeated the instruction to try not to move his/her head. We excluded a participant from the final analysis because the experimenter had to do this more than twice during the experiment. After each block, the experimenter entered the room and asked the participants how many faces with glasses (the correct answer was 8) appeared and what the models were doing. We did not analyze the number of faces with glasses because this question was only to ensure their attention was drawn to the eyes.

 The faces of the participants were recorded using a hidden video recorder, which was also used to monitor the participants. The videos were coded off-line, and the coder was blind to the stimulus the children were watching. Yawning was defined as the presence of the stereotyped motor pattern of gaping of the mouth accompanied by a long inspiration followed by a shorter expiration [5]. After coding, the number of yawns of participants in each block, the duration of each experimental block, and the eye-tracking data of the yawn block were analyzed. The duration of each experimental block differed between participants, as the presentation of each stimulus was dependent on participants' eye movements. A second coder also coded a subset of the video recordings (i.e., seventeen children), and the interrater concordance rate, which is the percentage of the cases where the numbers of yawns counted by two coders were the same, was 97%.

#### 2.1.4. Analyses

 Nonparametric tests were used to contrast the frequency of yawning (Wilcoxon signed-rank tests or Mann-Whitney tests, as appropriate) and the number of participants who yawned in each condition (*X* ^2^ tests), because the data was not normally distributed. For each of the analyses, we also confirmed any possible effect of IQ, gender, age, or ASQ-J score. We also analyzed the relationship between the frequencies of participants' yawning and eye-tracking measurements, again using nonparametric tests (see Supplementary Material for details).

### 2.2. Results

**Figure 3:**
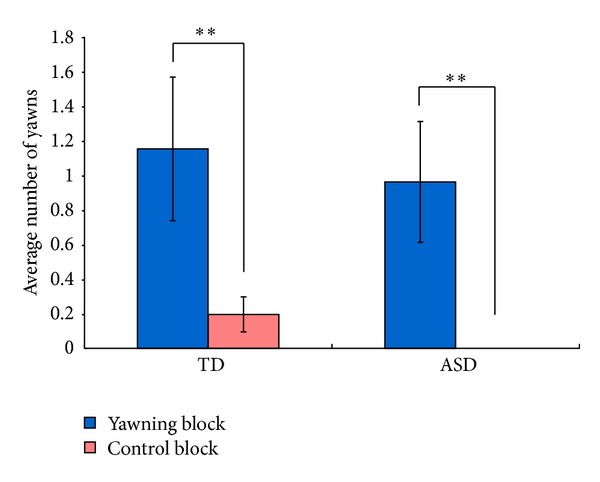
Average frequency of yawns of participants in yawn or control block in Experiment 1. TD: typically developing children; ASD: children with autism spectrum disorder; error bars: standard errors; **P < .01.

Figure 3 shows the average number of yawns of participants in each condition. On average, TD children yawned 1.2 times in the yawn condition and 0.2 times in the control condition. Children with ASD yawned 1.0 time in the yawn condition and did not yawn in the control condition. Both TD children and children with ASD yawned significantly more in the yawning block than in the control block (Wilcoxon signed-rank test: TD children: *Z* = −3.21, *P* < .01, *r* = −.47, children with ASD: *Z* = −2.69, *P* < .01, *r* = −.53). In addition, there were no differences between groups in the number of yawns in the yawning or in the control conditions (Mann-Whitney test: all |*Z*| < 1.54, all *P* > .12, all |*r*| < .19). The number of yawns in the yawning condition did not correlate with age, IQ, or scores of ASQ-J in either group (Spearman: all |*ρ*| < 0.28, all *P* > .17). The absence of correlation between the frequency of yawning and the age corroborates the above subgroup analysis to support the absence of age effect in the current age range tested. We also tested whether any gender difference in the frequency of yawning existed in the yawn and control conditions, but we found no significant gender difference in either TD children or children with ASD (Mann-Whitney test: all |*Z*| < 1.90, all *P* > .05, all |*r*| < .28).

We also conducted the analysis on the percentage of participants who yawned at least once in each condition. The percentage of participants who yawned at least once in the yawn condition was 35% in TD children and 35% in children with ASD. On the other hand, the percentage of participants who yawned at least once in the control condition was 8.7% in TD children and in none of children with ASD yawned during the control block. The percentages of participants who yawned at least once in the yawn condition were significantly higher than in the control condition both in TD children and children with ASD (TD children: *X* ^2^(1) = 9.2, *P* < .01, *φ* = .32, children with ASD: *X* ^2^(1) = 10.9, *P* < .01, *φ* = .46). We analyzed the effects of age, IQ, gender, and duration of the yawn block by dividing TD children and children with ASD of all ages into the “yawn group” and “no-yawn group” on the basis of whether they yawned or not in the yawn block. Both in TD children and in children with ASD, there was no significant group difference in chronological age (all |*t*| < 1.36, all *P* > .18, all *r* < .27) and IQ (all |*t*| < 0.55, all *P* > .59, all *r* < .09). We also tested whether any gender difference in the percentage of participants who yawned at least once existed. In TD children, we found no significant gender difference (the yawn condition: *X* ^2^(1) = 0.66, *P* = .42, *φ* = .12, the control condition: *X* ^2^(1) = 3.68, *P* = .06, *φ* = .28). We did not test this in children with ASD because there were only 6 females with ASD.

## 3. Experiment 2

### 3.1. Method

#### 3.1.1. Participants

The data of 29 TD participants (12 males and 17 females) and 22 participants with ASD (20 males and 2 females) were included in the final analyses (Table 2). Five more participants were also tested but not included in the analyses because their eye-tracking data did not provide valid gaze data in 50% or more observations during the period of stimulus presentation in a yawn block (a male participant with ASD), they were unable to answer the question about what the models were doing after the yawning block (2 male participants with ASD and a female participant with ASD), or they were moving excessively during the testing (a male participant with ASD). As in Experiment 1, most participants were recruited from Musashino Higashi Gakuen. All the children with ASD had been diagnosed with autism (*N* = 11), autism spectrum disorder (*N* = 1), Asperger's syndrome (*N* = 2), or pervasive developmental disorder (*N* = 8) by at least one child psychiatrist, clinical psychologist, or pediatrician before they participated in this experiment. The participants' parents all completed the Japanese version of the Autism Screening Questionnaire (ASQ-J [35, 36]). All ASQ-J scores of the TD children were below the cut-off point (13) and those of the children with ASD were at or above the cut-off point. The participants partially overlapped with those who participated in Experiment 1 (15 TD children and 9 children with ASD participated in both experiments), and Experiment 2 was conducted 1 year after Experiment 1.

There was no significant group difference in chronological age (*t*(34.4) = 1.66, *P* = .11, *r* = .27). On the other hand, there was also a significant group difference in sex ratio as in Experiment 1 (*X* ^2^(1) = 13.1, *P* < .01, *φ* = −.51). In the current study, we did not administer the Japanese WISC-III and WAIS-R because we found that IQ did not influence contagious yawning in Experiment 1.

#### 3.1.2. Stimuli

**Figure 4:**
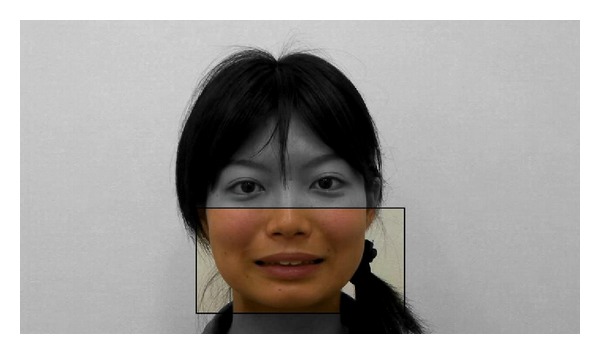
The mouth region of the stimuli: the stimuli started only when participants fixate within the black frame for at least 500 ms in Experiment 2.

 The stimuli were identical to those used in Experiment 1. Stimulus sequences were presented on a LCD monitor integrated with an eye tracker (Tobii 2150), which was placed about 60 cm from the participant. The eye tracker controlled the onset of stimuli (i.e., video clips of yawning and mouth-opening faces), which started only when participants fixated on the area around the mouth of the model for at least 500 ms, instead of the area around the eyes in Experiment 1. The face that appeared was oriented 25° vertically and 20° horizontally, and the region of the mouth was oriented 10° vertically and 20° horizontally (Figure 4), and the viewing distance was 60 cm. We also recorded the eye-tracking data during stimuli.

#### 3.1.3. Procedure

 The procedure in Experiment 2 was basically the same as in Experiment 1. All participants viewed the movies in a soundproofed room between about 9:30 and 18:30. They were asked to fixate on the mouth whenever faces appeared on the screen, instead of the eyes in Experiment 1. The participants were asked to count the number of times people with a beard appeared, in order to further ensure their attention was drawn to the mouth. After each block, the experimenter entered the room and asked the participants how many faces with a beard appeared and what the models were doing. The former question about a beard was used solely to ensure attention to the mouth area. Actually, the criteria for having a beard could be ambiguous in the current stimuli of Japanese adults, and thus, the correct answer was not set. We did not analyze the number of faces with a beard because this question was only to ensure their attention was drawn to the mouth.

 A second coder also coded a subset of the video recordings (i.e., thirteen children), and the interrater concordance rate was 96%.

#### 3.1.4. Analyses

 As in Experiment 1, nonparametric tests were used to contrast the frequency of yawning and the number of participants who yawned in each condition, and possible effects of IQ, gender, age, or ASQ-J score were analyzed. We also analyzed the relationship between the frequencies of participants' yawning and eye-tracking measurements using nonparametric tests (see Supplementary Material for details).

### 3.2. Results

**Figure 5:**
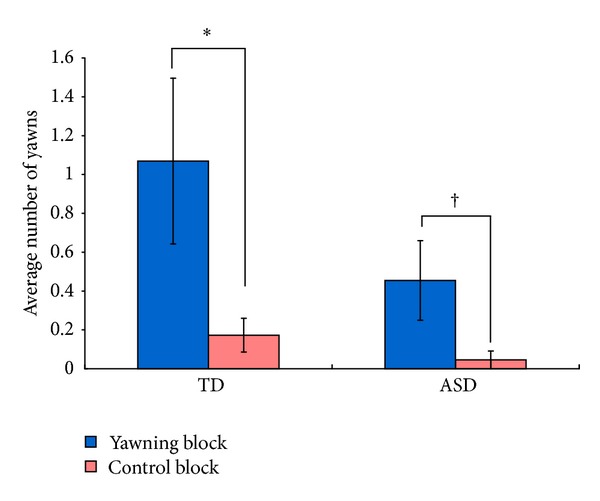
Average frequency of yawns of participants in yawn or control block in Experiment 2. TD: typically developing children; ASD: children with autism spectrum disorder; error bars: standard errors; *P < .05, ^†^ *P* < .10.

Figure 5 shows the average number of yawns of participants in each condition. On average, TD children yawned 1.1 times in the yawn condition and 0.2 times in the control condition. Children with ASD yawned 0.5 times in the yawn condition and yawned 0.04 times in the control condition. TD children yawned significantly more in the yawning block than in the control block, and children with ASD yawned marginally more in the yawning block than in the control block (Wilcoxon signed-rank test: TD children: *Z* = −2.05, *P* < .05, *r* = −.38, children with ASD: *Z* = −1.93, *P* < .10, *r* = −.41). In addition, there were no differences between groups in the number of yawns in the yawning or in the control conditions (Mann-Whitney test: all |*Z*| < 1.11, all *P* > .27, all *r* < .16). The number of yawns did not correlate with age or scores of ASQ-J in either group (Spearman: all |*ρ*| < 0.26, all *P* > .22). A subgroup of the participants who participated in both Experiments 1 and 2 were further analyzed to see if there were any systematic changes in yawning behaviour, but no significant differences were observed in either group (ASD, TD) or in either condition (yawning, control) (Wilcoxon signed-rank tests: all |*Z*| < 1.48, all *P* > .14, all *r* < .39). 

We also conducted the analysis on the percentage of participants who yawned at least once in each condition as in Experiment 1. The percentage of participants who yawned at least once in the yawn condition was 28% in TD children and 27% in children with ASD, and the percentage of participants who yawned at least once in the control condition was 14% in TD children and 4.5% in children with ASD. The percentage of participants who yawned at least once in the yawn condition was significantly higher than in the control condition in children with ASD; on the other hand, there was no difference in the percentage between two conditions in TD children (TD children: *X* ^2^(1) = 1.68, *P* = .20, *φ* = −.17, children with ASD: *X* ^2^(1) = 4.25, *P* < .05, *φ* = .31). 

We analyzed the effects of age, gender, and duration of the yawn block by dividing TD children and children with ASD of all ages into the “yawn group” and “no-yawn group” as in Experiment 1. Both in TD children and in children with ASD, there was no significant group difference in chronological age (all |*t*| < 1.00, all *P* > .33, all *r* < .22). We also tested whether any gender difference in the percentage of participants who yawned at least once existed. In TD children, we found no significant gender difference both in the yawn condition (*X* ^2^(1) = 2.03, *P* = .15, *φ* = .27) and in the control condition (*X* ^2^(1) = 2.16, *P* = .14, *φ* = .27). We did not test this in children with ASD because there were only 2 females with ASD. We also compared the duration of each experimental block between TD children and children with ASD and found that there was no difference in the duration of each experimental block (all |*t*| < 1.31, all *P* > .19, all *r* < .19). Moreover, there was no difference in the duration of each experimental block between the “yawn group” and the “no-yawn group” both in TD children and in children with ASD (all |*t*| < 1.09, all *P* > .29, all *r* < .35).

## 4. Discussion

 In both experiments, more children with ASD yawned in response to yawning stimuli than to control stimuli, which demonstrates that video images of yawning faces can elicit yawning in children with ASD, when the onset of a stimulus presentation is contingent on participants' fixation on the face. Around 30% of children with ASD showed contagious yawning, which is equivalent to the rates of contagious yawning in the control children and significantly more than those who yawned in response to the nonyawning stimuli. The rate of contagious yawning in the current study is well within the range of the rate of contagious yawning in other studies around the same age range (12–60% [12, 15, 16]). The results suggest that individuals with ASD do not have a fundamental impairment in catching others' yawns, such as the impairment to empathize with others [41]. Instead, it is possible that the previous finding that children with ASD were less susceptible to contagious yawning is modulated by the atypical development of spontaneous social attention to the face (e.g., [28, 29]). The current study corroborates previous findings that individuals with ASD can demonstrate behavioural contagion [42], attentional engagement [29], and partially normalized neural processing of the face [43–45] when the experimental control effectively navigates the attention of the participants to the face.

 The results do not fully support the special role of initial fixations on the eyes to elicit contagious yawning (e.g., [14]), because we observed contagious yawning when participants' attention was drawn to the mouth (Experiment 2). However, we emphasize that our results should not be taken as the evidence that observation of yawning eyes is irrelevant to contagious yawning for the following reasons. Firstly, our yawning stimuli lasted 5 seconds, which provides sufficient opportunity for the participants to saccade from the mouth to the eyes in Experiment 2. Secondly, initial attention to the eyes (Experiment 1) elicited twice as frequent yawning as the initial attention to the mouth (Experiment 2) in children with ASD, even though this difference did not reach statistical significance (Mann-Whitney test: *Z* = 0.78, *P* = .43). Further studies will be necessary to test the role of the pattern of face fixation on contagious yawning, especially in individuals with ASD.

 One limitation of the current study is that atypical fixation on the face cannot explain all previous reports of the absence of contagious yawning in ASD, because this absence has also been reported in response to yawning voices, where there was no visual presentation of yawning eyes [12]. Further studies, which do not involve visual stimuli, will therefore be required to study the role of atypical social orienting to the absence of contagious yawning in ASD. Note that the only neuroimaging study that has demonstrated activation of the mirror neuron system (i.e., inferior frontal cortex) used auditory stimuli [21], which might suggest that the mirror neuron system plays a critical role in contagious yawning when visual stimuli are not available. 

The current study has demonstrated that experimentally controlled fixations on yawning eyes can induce contagious yawning in individuals with ASD. The results suggest that contagious yawning requires attention to the yawning individuals, which could be affected in individuals with ASD. Further studies will be beneficial to investigate whether this is also the case for other clinical populations, such as individuals with schizophrenia, who also demonstrate an absence of contagious yawning [46]. It is important to explore the effect of induced contagious yawning on social cognition and behaviour in these clinical populations, which will help us understand the function of contagious yawning. Other important questions include whether individual differences in susceptibility to contagious yawning are related to an individual's tendency to spontaneously orient to others' faces and whether the relationship between contagious yawning and attention to others can be observed in nonhuman animals too. These studies will help to reveal the neural and cognitive mechanisms underlying contagious yawning, as well as its function, development, and evolution.

## Supplementary Material

The Supplementary Material contains additional analyses on the effect of the duration of the experiment, as well as the analyses of eye-tracking data.

## Figures and Tables

**Table 1:** Mean, standard deviation (SD) and range of chronological age, full intelligence quotient (FIQ), and scores on the Japanese version of the Autism Screening Questionnaire (ASQ-J) of participants in Experiment 1.

	Age (years)	FIQ	ASQ-J
TD (*n* = 46)			
M (SD)	11.2 (3.1)	105.5 (11.7)	2.3 (2.4)
Range	6.7–18.6	82–136	0–8
ASD (*n* = 26)			
M (SD)	12.4 (3.5)	91.6 (25.3)	22.7 (5.4)
Range	6.6–18.8	46–127	14–31

**Table 2:** Mean, standard deviation (SD) and range of chronological age, and scores on the Japanese version of the Autism Screening Questionnaire (ASQ-J) of participants in Experiment 2.

	Age (years)	ASQ-J
TD (*n* = 29)*		
M (SD)	12.7 (2.4)	2.3 (2.4)
Range	9.8–17.9	0–7
ASD (*n* = 22)*		
M (SD)	14.2 (3.7)	22.4 (4.7)
Range	7.7–19.8	14–31

*In Experiment 2, the Japanese WISC-III and WAIS-R were not administered because IQ did not influence contagious yawning in Experiment 1.
