# Supplementary material for: Presence of Contagious Yawning in Children with Autism Spectrum Disorder
Source: Autism Res Treat. 2013 Jul 22;2013:971686. doi: 10.1155/2013/971686 (PMC3736493; doi:10.1155/2013/971686)
Supplement: Supplementary file 1 — The Supplementary Material contains additional analyses on the effect of the duration of the experiment, as well as the analyses of eye-tracking data. [file 971686.f1.pdf]

Supplementary Results

Duration of experimental blocks

In Experiment 1, we also compared the duration of each experimental block between TD children and children with ASD, and found that the duration of the yawning block in children with ASD was significantly longer than in TD children ($t(32.9) = 2.04$, $p < .05$, $r = .34$) and that of the control block in children with ASD was marginally longer than TD children ($t(70) = 1.80$, $p = .08$, $r = .21$). Moreover, the duration of the yawning block in the "yawn group" (*i.e.* those who yawned at least once) was significantly longer than in the "no-yawn group" (*i.e.* those who didn't yawn) only in children with ASD (TD children: $t(19.2) = -1.68$, $p = .11$, $r = .36$, children with ASD: $t(8.6) = -2.51$, $p < .05$, $r = .65$), and the duration of the control block in the "yawn group" was significantly longer than in the "no-yawn group" both in TD children and children with ASD (TD children: $t(20.5) = -3.03$, $p < .01$, $r = .56$, children with ASD: $t(24) = -2.87$, $p < .01$, $r = .51$).

Thus, we also conducted the analysis on the number of yawns per minute in order to control for the duration of each experimental block. We found that the number of yawns per minute in the yawning condition was significantly more than that in the control condition in both TD children and children with ASD (Wilcoxon signed rank test: TD children: $z = -3.43$, $p < .01$, $r = -.51$, children with ASD: $z = -2.67$, $p < .01$, $r = -.52$). In addition, there were no differences between TD children and children with ASD in the number of yawns per minute in the yawning or in the control condition (Mann-Whitney test: the yawning condition: $z = 0.08$, $p = .93$, $r = .01$ the control condition: $z = 1.54$, $p = .13$, $r = .18$).

In Experiment 2, we found that the number of yawns per minute in the yawning condition was significantly more than that in the control condition in both

TD children and children with ASD (Wilcoxon signed rank test: TD children: $Z = -2.09$, $p < .05$, $r = -.39$, children with ASD: $Z = -2.20$, $p < .05$, $r = -.47$). In addition, there were no differences between TD children and children with ASD in the number of yawns per minute in the yawning or in the control condition (Mann-Whitney test: the yawning condition: $Z = 0.29$, $p = .77$, $r = .04$, the control condition: $Z = 1.16$, $p = .25$, $r = .16$).

Eye-tracking data analysis

In Experiment 1, we also analyzed group differences in eye-tracking data and their relationships with yawning. First, we calculated the percentage of the time the participant was looking at the eye region of stimuli, defined previously by dividing total looking time to the eye region by total looking time when the eye-tracking data was collected validly in the yawning block. This percentage in children with ASD was significantly lower than in TD children (TD children: mean = 86%, SD = 16%, children with ASD: mean = 74%, SD = 11%, $t(37.9) = -3.48$, $p < .01$, $r = .49$). This means that children with ASD were looking at the eye region less than TD children even though all participants were asked to fixate on the eyes whenever faces appeared on the screen. Second, we compared the "yawn group" with the "no-yawn group". Both in TD children and children with ASD, the percentage of time when they were looking at the eye region in the "yawn group" was significantly lower than in the "no-yawn group" (TD children: $t(44) = 3.00$, $p < .01$, $r = .41$, children with ASD: $t(24) = 3.58$, $p < .01$, $r = .59$). Also, there was a negative correlation between the percentage of time when they were looking at the eye region and the number of yawns in both TD children and children with ASD (Spearman: TD children: $\rho = -0.47$, $p < .01$,

children with ASD: ρ = -0.57, $p < .01$). There results might suggest that children who yawned wandered off from the task-relevant features as a result of their own yawns.

In Experiment 2, we also calculated the percentage of the time the participant was looking at the eye region (Figure 2) and the mouth region (Figure 4) of the stimuli respectively by dividing total time looking at the eye region and the mouth region by total looking time when the eye-tracking data was collected validly. There was no significant difference in these percentages between children with ASD and TD children (the eye region: TD children: mean = 47% (SD = 13%), children with ASD: mean = 52%, SD = 12%, $t(49) = 1.16$, $p = .25$, $r = .16$, the mouth region: TD children: mean = 72% (SD = 16%), children with ASD: mean = 70% (SD = 17%), $t(49) = -0.64$, $p = .53$, $r = .09$).

We also compared the percentages of time when they were looking at the eye region and the mouth region between the "yawn group" and the " no-yawn group". Both in TD children and children with ASD, there was no significant difference in the percentages when they were looking at the eye or the month region between the two groups (all $|t| < 1.73$, all $p > .10$, $r < .36$), and there was no correlation between the percentages and the number of yawns (all $|\rho| < 0.42$, $p > .05$).
